# Musical Intensity Applied in the Sports and Exercise Domain: An Effective Strategy to Boost Performance?

**DOI:** 10.3389/fpsyg.2019.01145

**Published:** 2019-05-15

**Authors:** Edith Van Dyck

**Affiliations:** Department of Art History, Musicology and Theatre Studies, Institute for Psychoacoustics and Electronic Music (IPEM), Ghent University, Ghent, Belgium

**Keywords:** music, sports, exercise, intensity, hearing loss, sound pressure level, performance, loudness

## Abstract

In the sports and exercise domain, it has been suggested that musical intensity might boost performance. Previous research revealed that pumping up the volume of music might increase running speed, grip strength, and choice reaction time while simultaneously decreasing time to exhaustion and level of perceived exhaustion. However, evidence is still scarce, experimental designs and tested groups vary significantly, and contradicting evidence exists as well. Yet, listening to high-intensity music could be a risky business and exercisers employing such a strategy to improve performance are vulnerable to developing noise-induced hearing problems. Therefore, future research should inquire more profoundly into the inherent boosting qualities of musical intensity and juxtapose experimental results and auditory repercussions in order to uncover possible strategies to combine both in such a way that the exerciser’s health can be safeguarded at all times.

In sports and exercise, music is regularly employed as a tool to accompany the exerciser’s movements and gestures. This makes sense, since previous research indicated that music listening during sports activities can capture attention (Priest and Karageorghis, 2008), distract from fatigue and discomfort (Yamashita et al., 2006), prompt and alter mood states (Edworthy and Waring, 2006; Shaulov and Lufi, 2009), enhance work output (Priest et al., 2004; Rendi et al., 2008), increase arousal (Lim et al., 2014), relieve stress (Särkämö et al., 2008), stimulate rhythmic movement (Atkinson et al., 2004), and evoke a sense of power and produce power-related cognition and behavior (Hsu et al., 2014). As such, a musical accompaniment can be a valuable commodity to boost the personal state of the exerciser as well as his/her performance.

Naturally, the obtained effects on performance output depend on features of the particular stimulus, the exerciser, the intensity of the activity, etc. To exemplify, it was indicated that up-tempo music could have enhancing effects on performance, while slow-tempo music might obtain relaxing or detrimental reactions (Tenenbaum et al., 2004; Edworthy and Waring, 2006; Rendi et al., 2008; Waterhouse et al., 2010; Van Dyck et al., 2015). Musical features such as the meter (Leman et al., 2013) or the level of synchronicity with the activity (Simpson and Karageorghis, 2006; Terry et al., 2012) were also shown to impact performance. Next to stimulus-related features, subject-related characteristics were also demonstrated to play a role. The personal taste of the exerciser carries additional weight, since music perceived as demotivating or rated low regarding the individual’s preference might only impact the achievement to a minor extent or simply prove to be counterproductive (Terry et al., 2012; Hutchinson et al., 2018). Another important parameter regards the intensity of the activity. Physiological cues are likely to dominate the exerciser’s processing capacities when exercising at high intensity levels, while at moderate levels, musical and physiological cues can be processed in parallel. In other words, when the workload becomes too high, the exerciser’s attention is typically shifted toward the painful or fatiguing effects of the exercise, thus resulting in an impact reduction of the musical effects (Nethery, 2002; Hutchinson and Tenenbaum, 2007; Razon et al., 2009; Lima-Silva et al., 2012).

It could be questioned if either of the abovementioned or related properties might provoke adverse effects. People generally exercise to improve their health, thus possible negative reverberations could be of crucial interest to the exerciser. In the light of this special issue, the aim is to discuss the *intensity* (or *volume*, *sound pressure level*), as well as the subjective perception (*loudness*), of musical stimuli implemented in the sports and exercise domain. In sports science, much attention has been directed toward several aspects of the musical accompaniment, primarily focusing on tempo (e.g., Karageorghis et al., 2006, 2008), while only limited attention was paid to the intensity of the music (Hutchinson and Sherman, 2014). In the further course of this review, a light is shed on its possible impact and related implications in sports and exercise.

## Musical Intensity as a Tool to Boost Performance: Evidence and Mechanisms

### Evidence

Some studies set out to assess the impact of musical intensity on exercisers’ performance. Such research was performed in a range of areas, mainly—but not exclusively—focusing on cyclic sports activities such as running and cycling, and often exploring different musical features simultaneously. An investigation into the available material promptly uncovered a heterogeneous body of employed methodologies and a disparity between the results and conclusions of the performed research.

In the area of auditory warning design, researchers generally agree on the capacity of louder auditory warnings to produce faster reaction times and retain certain physiological correlates (Burt et al., 1995; Haas and Casali, 1995; Haas and Edworthy, 1996; Brown et al., 2008). Moreover, greater loudness can positively influence referees’ judgments in team sport games (Unkelbach and Memmert, 2010). In line with the aforementioned, Copeland and Franks (1991) measured heart rate, rating of perceived exertion (RPE), and time to exhaustion during a treadmill experiment in three different conditions: soft, slow, easy-listening music; loud, fast popular music; and no music. Results indicated that heart rate, time to exhaustion, as well as level of perceived exhaustion were lower for the slow, soft music compared to the loud, fast stimuli. Correspondingly, Edworthy and Waring (2006) tested the effect of fast/loud, fast/quiet, slow/loud, slow/quiet music, or silence on running speed, heart rate, perceived exertion, and affect during a 10-min treadmill exercise. Results showed that the speed of running was particularly affected by music tempo, while increased volumes did not produce faster speeds. Yet, an interaction effect was retrieved, demonstrating the highest impact on running speed for loud, fast music. Additionally, heart rate increased after the introduction of louder fast musical excerpts, but not when the volume of slow music was increased.

In the abovementioned research, music was presented during the execution of the activity under study. Yet, pretask music is widely used by athletes as well. Karageorghis et al. (2018) explored the impact of tempo and intensity of music presented prior to the exercise, focusing on grip strength and subjective affect (valence and arousal). During the experiments, male athletes were exposed to five conditions: fast/loud [126 BPM/80 dB(A)], fast/quiet [126 BPM/70 dB(A)], slow/loud [87 BPM/80 dB(A)], slow/quiet [87 BPM/70 dB(A)] music, and a no-music control. Again, interaction effects of the tempo and intensity components were retrieved for the performance feature. The highest levels of grip strength were retrieved when fast music was played loudly. Up-tempo music presented at lower intensities, however, yielded significantly lower grip strength performance. Similarly, in a study regarding tennis players’ choice reaction time (CRT), music played at a fast tempo and high intensity was shown to yield shorter reaction times in a subsequent CRT task than did fast, moderate-intensity music (Bishop et al., 2009). Interestingly, it was pointed out that such effects are overall more pronounced for untrained or recreational exercisers compared to (professional) athletes (Mohammadzadeh et al., 2008; Karageorghis and Priest, 2012; Hutchinson and Sherman, 2014). These results seem to indicate that the intensity of a musical stimulus – particularly in interaction with the tempo and when used by recreational exercisers – can be employed to enhance optimal exercising in a range of sports activities.

### Mechanisms

As has been evidenced repeatedly, preference is key when regarding the influence of musical stimuli on human behavior in general as well as when considering exercise performance, and thus interacts with intensity selection (Karageorghis and Priest, 2012). Some have indicated that the more listeners like a certain stimulus, the louder they wish to listen to it (Cullari and Semanchick, 1989). In the same line, Fucci (1993) demonstrated that, when a number of musical excerpts are played at the same sound pressure level, well-liked fragments can be perceived as less loud compared to the lesser preferred. In research by Karageorghis et al. (2018), main effects of the intensity of pretask music on affective valence were retrieved, suggesting that athletes perceive loud music as more pleasurable compared to less intense sounds. Production and dissemination strategies in the music and broadcasting industries seem to agree with the notion of a general preference for loud stimuli (Vickers, 2010; Halevi-Katz et al., 2015). According to Todd and Cody (2000), acoustically evoked sensations of self-motion might account for the compulsion to exposure to loud music. They revealed that high volume levels of dance music [above 90 dB(A) SPL] were associated with vestibular responses to low-frequency beats. As such, the researchers assumed that these responses could reach the pleasure centers of the brain via the thalamus and advocated that this may be a physiological basis for the minimum intensity level of music in order to be regarded as “pleasurable” by the listener. Furthermore, a maladaptive pattern of music-listening behavior similar to that of substance users was put forward, suggesting that loud music listening might be addictive to some (Florentine et al., 1998; Carlson et al., 2015). Music listening at high intensities does indeed share three pivotal elements of substances triggering patterns of addictive behavior: (1) the capacity to induce rapid and potent changes in mood and level of arousal; (2) the ability to reduce negative mood states; and (3) the tendency to elicit the experience of craving (Donovan, 1988).

Loud music might particularly affect human behavior due to its arousing properties. Arousal refers to an enhanced physiological state where our body and senses are alert and active while our emotions are intensified (Welch and Fremaux, 2017a). Increasing sound pressure levels leads to intensified arousal, which may be of substantial importance to sports and exercise performance (Edworthy and Waring, 2006). In their study on grip strength, Karageorghis et al. (2018) failed to detect differences between musical tempi for reported levels of arousal, yet the high-intensity condition of the experiment did prove to yield elevated arousal scores. Bishop et al. (2009) retrieved similar effects regarding tennis players’ CRT performance, with higher volumes indicating to amplify arousal levels. In addition, musical intensity might also facilitate to distract attention from bodily perceptions to external cues (e.g., Hutchinson and Sherman, 2014; Murgia and Galmonte, 2015). This is especially relevant in the sports domain, as feelings of fatigue or discomfort can be facilitated by the use of more dominantly present stimuli. This is at least the case up to a certain level of exercise intensity (when the intensity increases up to a point where distraction from internal sensations of fatigue can no longer be achieved), often referred to as the *ventilatory threshold* (Karageorghis and Terry, 1997).

Welch and Fremaux (2017a) looked beyond the mere *intrapersonal-level influences* of arousal and also stressed the prominence of *cultural-level influences* (the expectation of loudness that is shaped by previous encounters) as well as *interpersonal-level influences* (the shared experience of the music dominating the environment, enabling a group to experience a similar state and thus draw people together). They also presented a theoretical basis for the processing behind the urge of some to expose themselves to loud music (either in individual or social contexts). Four main themes relating to the enjoyment of loud sound were stressed in the Conditioning, Adaptation and Acculturation to Loud Music (CAALM) Model: (1) an initial physiological adaptation enabling people to overcome the possible discomfort of loud music, (2) a conditioning response of repeated pairing of loudness with perceived benefits of high-volume music itself (e.g., masking, social benefits, arousal, excitement) and other commonly associated advantages (e.g., performance output, fun, friends, etc.), and (3) an acculturation process wherein large groups of people start to regard loud music as the norm and relate it to positive ways of expressing themselves (Welch and Fremaux, 2017b).

Although the above examples and the underlying mechanisms might imply possible (main and interaction) effects of musical intensity on performance, findings of some recent experimental research tend to be at odds. Metcalfe et al. (2016) enquired into the influence of intensity variations [45 and 75 dB(A)] in background music on walking behavior but failed to demonstrate any effects. Similarly, Kreutz et al. (2018) regarded the impact of electronic music at different intensity levels [0, 65, 75, and 85 dB(A)] on ergometer performance (i.e., physical performance, force on the pedal, and pedaling frequency), perceived fatigue, and heart rate in healthy adults with different training levels. Although, based on previous research, it was hypothesized that higher intensity levels of the sound would be associated with greater ergometer performance and less perceived effort, no such effects were retrieved in the course of the study. Therefore, the researchers suggested that the high acceptance of loud music and perceived appropriateness might rely on erroneous beliefs or stereotypes and, in their opinion, the reduction of musical intensity during sports and exercise may not compromise physical performance or perceived effort.

Although most performed research on the topic hinted at a tenable effect of the intensity of exercise-supporting stimuli, until further notice, such findings cannot be generalized. Research on the matter is still scarce and only covers a limited area of specific sports disciplines. Employed methodologies often contrast as well; musical stimuli and tested populations strongly vary between experiments, while such choices alone might determine the test output. Another important parameter is the duration of the test phase, a feature that might account for the disparity in obtained results of previous studies. In the experiment performed by Kreutz et al. (2018) for example, ergometer performance was tested for 45 minutes continuously. Conversely, in the case of Edworthy and Waring (2006), a 10-min treadmill exercise was performed. Although the exact duration of the test phase in the studies of Bishop et al. (2009) and Karageorghis et al. (2018) varied, all experiments were significantly shorter in time compared to the one performed by Kreutz et al. (2018). Besides, Copeland and Franks (1991) specifically trialed their running/walking participants until voluntary exhaustion. As such, it might be suggested that musical intensity effects are either rather immediate or that a habituation effect emerges after a certain period of time, resulting in a reduced or even completely absent impact of the sound pressure level over time. This might also relate to the previously discussed ventilatory threshold (Karageorghis and Terry, 1997), as the exercise intensity could become too strenuous after a certain amount of time, disabling the exercisers to further focus on the musical stimulus, which therefore loses its power to distract, motivate, arouse, and/or boost performance.

## Musical Intensity and Leisure: Less is More

One could question whether these debated effects of musical intensity might prevail over the potential detrimental influences regarding the hearing capacities of the listeners. Evidently, listening to high-intensity music while exercising is not without risk. Millions of adolescents and young adults are at risk of developing hearing loss because of their frequent exposure to loud music during leisure activities (Zhao et al., 2010; Carter et al., 2014; WHO, 2015). In fitness sports, for example, the use of high-intensity music is widespread (Kreutz et al., 2018). In a study enquiring into an aerobic endurance tournament, measured intensity levels ranged from 101 to 119 dB(A) during the 120-min duration of the activity (Chacón Araya and Moncada Jiménez, 2008). Others have underscored that, since high-intensity music listening in aerobic classes was shown to relate to enjoyment and motivation to work, exercisers do generally not regard it as dangerously loud. As such, aerobics and variant fitness activities become at-risk activities for noise-induced hearing loss (Yaremchuk and Kaczor, 1999; Nassar, 2001; Wilson and Herbstein, 2003). This notion has also been supported by the trainers themselves, with aerobics/fitness instructors complaining about fluctuating hearing loss, tinnitus, and other auditory problems from their profession (Yaremchuk and Kaczor, 1999; Beach and Nie, 2014; Nie and Beach, 2016). Consequently, fitness studio attendance has been explicitly included in a portfolio of potentially harmful activities for adolescents’ and young adults’ hearing (Beach et al., 2013).

Besides, the use of personal listening devices, of which maximum output levels often exceed safety barriers (Fligor and Cox, 2004; Peng et al., 2007; Portnuff et al., 2011), during sports activities is widespread, especially for activities such as running and cycling. Yet, it was estimated that nearly 50% of people aged between 12 and 35 years are exposed to dangerously elevated levels of music through their personal listening devices (WHO, 2015). Research by Keppler et al. (2010) evidenced that after only an hour of pop/rock music listening through an MP3 player, young participants already experience temporary hearing loss. Nowadays, music is available at all times and smartphones are accompanying us wherever we go, even to the gym or running track, while downloading and streaming possibilities are towering. As a consequence, owning a smartphone might imply a substantial level of vulnerability when no precautions are taken (Shim et al., 2018).

When assessing adolescents and young adults’ opinion regarding sound pressure levels at music events, it was evidenced that this population in fact does not demand excessive noise levels. Moreover, most young people tend to show a positive attitude toward more strict noise regulations (Mercier and Hohmann, 2002; Gilles et al., 2014). Yet, most adolescents and young adults share a fairly positive or neutral way of thinking regarding excessive noise exposure, indicating that they hardly perceive it as a risk, but rather as something ordinary in today’s society (Gilles et al., 2013; Landälv et al., 2013). In the sports and exercise domain, prevention campaigns focusing on the risks of highly amplified music hardly exist, while in the clubbing scene, such strategies are implemented habitually (Gilles and Van de Heyning, 2014). As previous research demonstrated that the model of the theory of planned behavior might provide a theoretical framework to predict and alter human health behavior (Weichbold and Zorowka, 2005), employing preventive campaigns might amend exercisers’ attitudes toward noise and hearing protection (Gilles and Van de Heyning, 2014).

## Conclusion

In the sports and exercise domain, previous research examined whether musical intensity might impact performance output, displaying contrasting results. Some indicated that the sound pressure level of a musical stimulus could successfully be employed to enhance optimal exercising, while others refuted such effects. Clearly, more research on the topic (e.g., on the intensity and duration of the boosting effect, regarding a wider scope of exercise activities, comparing recreational and professional exercisers, taking music preference into account, etc.) is required in order to formulate straightforward recommendations regarding the use of loud music as a means to boost performance. Yet, even if a distinct impact on specific activities might be validated, one should still remain cautious. Intensity strongly differs from other musical features (e.g., tempo, tonality, etc.) in the fashion that it might prompt strong adverse effects regarding hearing capabilities. With respect to sports and exercise, evidence demonstrated that listeners generally fail to consider high-intensity music to be too loud if they find it enjoyable and/or motivating, which might be even further amplified in case of apparent improvements in exercise performance. Such potential benefits can be rather immediate while the possible implications for the individual may reveal themselves only much later.

## Author Contributions

The author confirms being the sole contributor of this work and has approved it for publication.

### Conflict of Interest Statement

The author declares that the research was conducted in the absence of any commercial or financial relationships that could be construed as a potential conflict of interest.
