# Corrigendum: Musical Intensity Applied in the Sports and Exercise Domain: An Effective Strategy to Boost Performance?

**DOI:** 10.3389/fpsyg.2019.01434

**Published:** 2019-06-20

**Authors:** Edith Van Dyck

**Affiliations:** Department of Art History, Musicology and Theatre Studies, Institute for Psychoacoustics and Electronic Music (IPEM), Ghent University, Ghent, Belgium

**Keywords:** music, sports, exercise, intensity, hearing loss, sound pressure level, performance, loudness

In the original article, there was an error. In the text, it reads “Bishop et al. (2009) retrieved similar effects regarding tennis players' CRT performance, with lower volumes indicating to amplify arousal levels.”

Yet, the correct text is “Bishop et al. (2009) retrieved similar effects regarding tennis players' CRT performance, with higher volumes indicating to amplify arousal levels.”

The author apologizes for this error and state that this does not change the scientific conclusions of the article in any way. The original article has been updated.
